# Comprehensive analyses of the ARF gene family in cannabis reveals their potential roles in regulating cannabidiol biosynthesis and male flower development

**DOI:** 10.3389/fpls.2024.1394337

**Published:** 2024-06-05

**Authors:** Gen Pan, Xiaojuan Yang, Jiajia He, Zhenyi Liu, Fengming Chen, Jiayi Chen

**Affiliations:** ^1^ Hunan Provincial Key Laboratory of the Traditional Chinese Medicine Agricultural Biogenomic, Changsha Medical University, Changsha, China; ^2^ Institute of Chinese Medicine Resources, Hunan Academy of Chinese Medicine, Changsha, China; ^3^ Institute of Bast Fiber Crops, Chinese Academy of Agricultural Sciences, Changsha, China

**Keywords:** cannabis, auxin response factor, male flower development, cannabidiol biosynthesis, CBD yield

## Abstract

**Background:**

Cannabidiol (CBD), as an important therapeutic property of the cannabis plants, is mainly produced in the flower organs. Auxin response factors (ARFs) are play a crucial role in flower development and secondary metabolite production. However, the specific roles of ARF gene family in cannabis remain unknown.

**Methods:**

In this study, various bioinformatics analysis of *CsARF* genes were conducted using online website and bioinformatics, quantitative real time PCR technology was used to investigate the expression patterns of the *CsARF* gene family in different tissues of different cannabis varieties, and subcellular localization analysis was performed in tobacco leaf.

**Results:**

In this study, 22 *CsARF* genes were identified and found to be unevenly distributed across 9 chromosomes of the cannabis genome. Phylogenetic analysis revealed that the ARF proteins were divided into 4 subgroups. Duplication analysis identified one pair of segmental/whole-genome duplicated *CsARF*, and three pairs of tandemly duplicated *CsARF*. Collinearity analysis revealed that two *CsARF* genes, *CsARF4* and *CsARF19*, were orthologous in both rice and soybean. Furthermore, subcellular localization analysis showed that *CsARF2* was localized in the nucleus. Tissue-specific expression analysis revealed that six genes were highly expressed in cannabis male flowers, and among these genes, 3 genes were further found to be highly expressed at different developmental stages of male flowers. Meanwhile, correlation analysis between the expression level of *CsARF* genes and CBD content in two cultivars ‘H8’ and ‘Y7’ showed that the expression level of *CsARF13* was negatively correlated with CBD content, while the expression levels of six genes were positively correlated with CBD content. In addition, most of *CsARF* genes were responsive to IAA treatment.

**Conclusion:**

Our study laid a foundation for the further studies of CsARFs function in cannabis, and provides candidate genes for breeding varieties with high CBD yield in cannabis production.

## 1 Introduction

Cannabis is an ancient economic crop that generally includes both female and male plants in an approximate ratio of 1:1 and is widely used in textiles, food, building materials, and other applications (Rana and Choudhary, 2010; Andre et al., 2016). It is a complex plant containing more than 500 chemicals, of which 125 are cannabinoids represented by cannabidiol (CBD) (Khalsa et al., 2022). CBD has physiologically active functions such as pain relief and anti-inflammatory, anti-epileptic and anti-insomniac effects, and has recently been widely used in the fields of pharmaceuticals, health products, cosmetics and forage (Salami et al., 2020). Cannabis seeds are a traditional Chinese medicine rich in proteins and oils (Crocq, 2020). Female cannabis flowers, which are the only organ used to harvest cannabis seeds, generally accumulate more CBD than other tissues of the plant. To produce more CBD and harvest more seeds, the proportion of female plants must be increased (Jiang et al., 2022). Additionally, as pollination of female plants by male plants reduces the content of CBD (Lipson et al., 2021), the proportion of male plants also should be decreased. Therefore, considering its important role in the quality of cannabidiol content and seed production, understanding the molecular mechanisms of flower development and CBD biosynthesis in cannabis is essential for optimizing its production.

CBD is a secondary metabolite produced and accumulated in glandular trichomes, and its synthetic pathway is relatively clear (Van Bakel et al., 2011; Hurgobin et al., 2021). The precursor synthesis of CBD occurs via two metabolic pathways: the polyketide pathway and the methylerythritol phosphate pathway. These two metabolic pathways produce two types of alkylresorcinolic acids, olivetolic acid (OA) and geranyl diphosphate (GPP), respectively. Subsequently, OA and GPP synthesize cannabidiolic acid (CBGA), the acidic precursor of CBD. Finally, CBGA undergoes further decarboxylation under physical conditions such as light and heat to generate CBD (Van Bakel et al., 2011). Many key enzyme genes involved in the CBD synthesis pathway have been identified, including those encoding acyl-activating enzymes (*AAE*), cannabidiolic acid synthase (*CBDAS*), lipoxygenase (*LOX*), and prenyltransferase (*PT*) (Van Bakel et al., 2011). Tissue-specific expression analysis revealed that the expression patterns of transcription factors were positively correlated with those of enzyme genes of the CBD synthesis pathway (Van Bakel et al., 2011), indicating that CBD biosynthesis may be regulated by some transcription factors. Though MYB transcript factor has been identified as the potential regulator for CBD biosynthesis (Liu et al., 2021), knowledge of the regulation mechanism of CBD biosynthesis in cannabis is fairly limited.

ARF, a set of transcription factor, functions in the auxin signaling pathway (Guilfoyle and Hagen, 2007). ARF proteins include three domain subunits, an amino-terminal DNA-binding domain, a conserved carboxy-terminal dimerization domain, and a non-conserved middle domain (Guilfoyle and Hagen, 2007). Among the three domains, the DNA-binding domain, found on the N-terminal region, can bind specifically to an auxin response element (AuxRE) TGTCNN in promoters to regulate the auxin gene expression (Li et al., 2016). With genome sequencing, more ARF family genes have been identified in plants (Li et al., 2023). For example, 23 ARF genes have been identified in Arabidopsis (Okushima et al., 2005), 25 in rice (Wang et al., 2007), 19 in grape (Wan et al., 2014), 31 in Chinese cabbage (Mun et al., 2012), 20 in barley (Tombuloglu, 2019), and 19 in pepper (Zhang et al., 2017). In *Arabidopsis thaliana*, auxin response factor mutant *arf8* plants exhibit delayed stamen development and limited growth of Arabidopsis petals (Nagpal et al., 2005; Varaud et al., 2011). However, a comprehensive analysis of the function of the *ARF* gene family in cannabis is lacking. In the present study, to identify the potential role of ARF genes in male flower development and the regulation of CBD biosynthesis, we performed a systematic analysis of the *ARF* gene family in cannabis using bioinformatics and investigated their temporal and spatial expression patterns. Thus, the results presented in this study provide a biological basis for further studies by analyzing the molecular functions of the *CsARF* genes family in cannabis.

## 2 Materials and methods

### 2.1 Identification and analysis of physical and chemical properties of the *CsARF* gene family members in cannabis

The protein sequences of 22 Arabidopsis ARF were downloaded from the Arabidopsis Information Resource (TAIR) (http://www.aabidopsis.org/). The cannabis genome, protein, and genome annotation files (GCA_900626175.2) were obtained from the NCBI for Biotechnology Information database (https://www.ncbi.nlm.nih.gov/) (Laverty et al., 2019). To obtain the ARF family candidate genes in the cannabis genome, 22 Arabidopsis ARF proteins were used as a query for comparison with the cannabis protein database by BLAST sequence alignment (E-value <1E^-5^) performed using the TBtools software (Chen et al., 2020). Next, the protein sequences encoded by the ARF family candidate genes were submitted to the NCBI Conserved Domain Database (CDD, https://www.ncbi.nlm.nih.gov/cdd/) to examine whether they contained the conserved ARF (AUX_RESP) and B3 DNA-binding domains. Various physicochemical parameters of the *ARF* genes were analyzed using ProtParam (http://web.expasy.org/protparam/), and their subcellular locations were predicated by Plant-mPLoc (http://www.csbio.sjtu.edu.cn/bioinf/Cell-PLoc-2/).

### 2.2 Phylogenetic analysis of CsARF proteins, and gene structure analysis of *CsARF* genes

MEGA7.0 was used to construct a phylogenetic tree using the neighbor-joining method, which was further edited using FigTree software. MEME software (http://meme-suite.org) was used to analyze the conserved motifs in the *CsARF* genes. The coding sequence and untranslated region (UTR) of *CsARFs* were extracted from the cannabis genome annotation file using TBtools software, which was also used to combine the gene conserved motif, coding sequence and UTR.

### 2.3 *Cis*-regulatory element analysis

TBtools software was used to extract the 2000 bp sequence upstream of the upstream transcription start site of the 22 *CsARF* genes (Chen et al., 2020), and then these sequence were applied to analyzed *cis*-regulatory elements using the PlantCARE database (http://bioinformatics.psb.ugent.be/webtools/plantcare/html/).

### 2.4 Chromosome distribution and synteny analysis of *CsARF*

Information on the chromosomal location of the *CsARF* genes was extracted from the cannabis genome file and gene annotation file using TBTools. Next, the physical location of the *CsARF* genes on the chromosomes was determined using TBtools. TBtools, MCscanX and Circos were used to calculate and draw collinear genes in the cannabis genome and among the different species.

### 2.5 Subcellular localization of CsARF2 in tobacco leaf cells

The coding sequence (CDS) of *CsARF2* and *AtARF6* was amplified using the ‘H8’ leaf and the ‘Col’ leaf cDNA as templates, respectively. After PCR amplification and detection by gel electrophoresis, the target sequence was recovered and ligated to the linearized pBI121-GFP vector. The recombinant plasmid was then transferred in T1 cell, and the positive clones were further confirmed by PCR.

The recombinant vector was transformed into GV3101 cell, and the positive clones confirmed by PCR amplification were incubated in LB medium and shaken until the OD value of the bacterial solution is 0.6, and it was further collected by centrifugation at 5000 r/min for 10 min. The bacteria solution was then suspended twice into the MS liquid medium containing 10 mM MES, 150 μM acetosytingone and 10 mM MgCl_2._ After standing for 3 h at room temperature, it was injected into tobacco leaves with a 1 mL needle and observed in laser scanning confocalmicroscopy (LSM880, Zesiss) after 48 h.

### 2.6 Plant materials and treatment conditions

Seeds of the ‘H8’ variety were sown in a greenhouse at the Institute of Bast Fiber Crops at the Chinese Academy of Agricultural Sciences. Greenhouse growth conditions have been previously reported (Jiang et al., 2022). Auxin (indole-3-acetic acid, IAA) (coolaber, Beijing, China) was dissolved in ethanol and diluted to the required concentrations (20 µM). After spaying with IAA, the leaves of ‘H8’ were sampled at 0, 1, 6 and 12 h for RNA extraction. Once the plant entered the reproductive stage, tissues including male flowers at different stages, roots, stems and leaves collected from male plants of the ‘H8’ variety were used for RNA extraction.

### 2.7 Determination of CBD content of cannabis male and female flowers

The two varieties, ‘H8’ and ‘Y7’, were grown in the greenhouse, and the growth conditions have been previously reported (Jiang et al., 2022). When the plants were at the flowering stage in the greenhouse, the CBD content of male and female flowers was measured as previously reported (Mandrioli et al., 2019).

### 2.8 RNA extraction and real-time quantitative polymerase chain reaction

Total RNA was extracted using EASYspin Plus Plant RNA Kit (Aidlab Biotechnologies Co., Ltd., Beijing, China) according to previously published protocols (Zhu et al., 2020). After assessing RNA quality on agarosegels, reverse transcription were performed according to the instructions of the Evo M-MLV RT Premix for qPCR Kit (Accurate, Changsha, China), and the SYBR^®^ Green Premix Pro Taq HS qPCR Kit (Accurate, Changsha, China) was used for real-time quantitative reverse transcription PCR (qRT-PCR) according to published protocols (Zhou et al., 2022). The primers used for qRT-PCR analysis are listed in **Supplementary Table S1**. Data analysis was performed using the 2^−ΔΔCt^ method.

### 2.9 Statistical analysis

Statistical analysis was performed using SPSS 18.0 software. Values are the mean and SD of three independent experiments with three replicates each. For statistical analysis, data were analyzed by one-way analysis of variance (ANOVA), and different letters indicate significant difference at *P* < 0.05. The correlation coefficient between the expression levels of genes and CBD content was analyzed *via* Pearson’s correlation coefficient and Student’s *t*-test.

## 3 Results

### 3.1 Identification of 22 *CsARF* genes in cannabis genome

We used 22 Arabidopsis ARF proteins as a query for BLAST with the cannabis protein database and identified 46 *ARF* candidate genes. After examining the conserved domains using the NCBI CDD databases, 22 cannabis ARF proteins were obtained. Their physicochemical properties were analyzed using ProtParam (http://web.expasy.org/protparam/). As shown in **Table 1**, the lengths of the CsARF proteins ranged from 353 (CsARF22) to 1147 (CsARF9) aa, molecular weights ranged from 39.62 to 128.29 kDa, pI varied from 5.25 to 9.46, the grand average of hydropathicity ranged from -0.738 to -0.408, and the aliphatic index varied from 65.25 to 81.14 (**Table 1**).

**Table 1:** Basic information of the ARF gene family in cannabis.

Gene	Gene ID	Length(aa)	MW(Da)	pl	GRAVY	Aliphatic index	Subcellular localization
CsARF1	LOC115694956	402	45497.57	9.46	-0.470	81.14	Nucleus
CsARF2	LOC115694957	402	45524.57	9.41	-0.479	80.42	Nucleus
CsARF3	LOC115694964	401	45399.42	9.41	-0.468	81.10	Nucleus
CsARF4	LOC115697396	750	82070.04	6.75	-0.421	65.25	Nucleus
CsARF5	LOC115697832	917	102087.75	6.15	-0.499	71.34	Nucleus
CsARF6	LOC115699321	395	43386.10	9.18	-0.553	71.09	Nucleus
CsARF7	LOC115699476	976	108735.84	6.12	-0.678	68.12	Nucleus
CsARF8	LOC115701709	951	105248.80	5.25	-0.482	72.85	Nucleus
CsARF9	LOC115705617	1147	128291.77	6.17	-0.738	66.93	Nucleus
CsARF10	LOC115705675	828	92233.62	6.30	-0.605	64.98	Nucleus
CsARF11	LOC115705687	826	91981.35	6.36	-0.596	65.25	Nucleus
CsARF12	LOC115705861	687	76724.49	5.85	-0.422	74.05	Nucleus
CsARF13	LOC115706063	666	74493.37	6.69	-0.492	72.00	Nucleus
CsARF14	LOC115706103	651	72629.94	5.94	-0.408	75.45	Nucleus
CsARF15	LOC115709608	1127	124856.20	6.42	-0.646	67.68	Nucleus
CsARF16	LOC115709736	701	76950.84	6.73	-0.416	69.02	Nucleus
CsARF17	LOC115714321	675	75399.84	5.96	-0.533	67.39	Nucleus
CsARF18	LOC115716126	661	74046.89	5.85	-0.578	68.68	Nucleus
CsARF19	LOC115718756	640	70503.68	5.45	-0.461	77.91	Nucleus
CsARF20	LOC115719139	799	88468.57	6.84	-0.448	72.52	Nucleus
CsARF21	LOC115719255	718	79055.78	7.31	-0.453	65.60	Nucleus
CsARF22	LOC115725443	353	39628.85	6.53	-0.454	76.86	Cytoplasm

ARF is the auxin response factor, MW is the molecular weight, and pI is the isoelectric point.

### 3.2 Gene structure and phylogenetic relationship of *CsARF* genes

**Figure 1:**
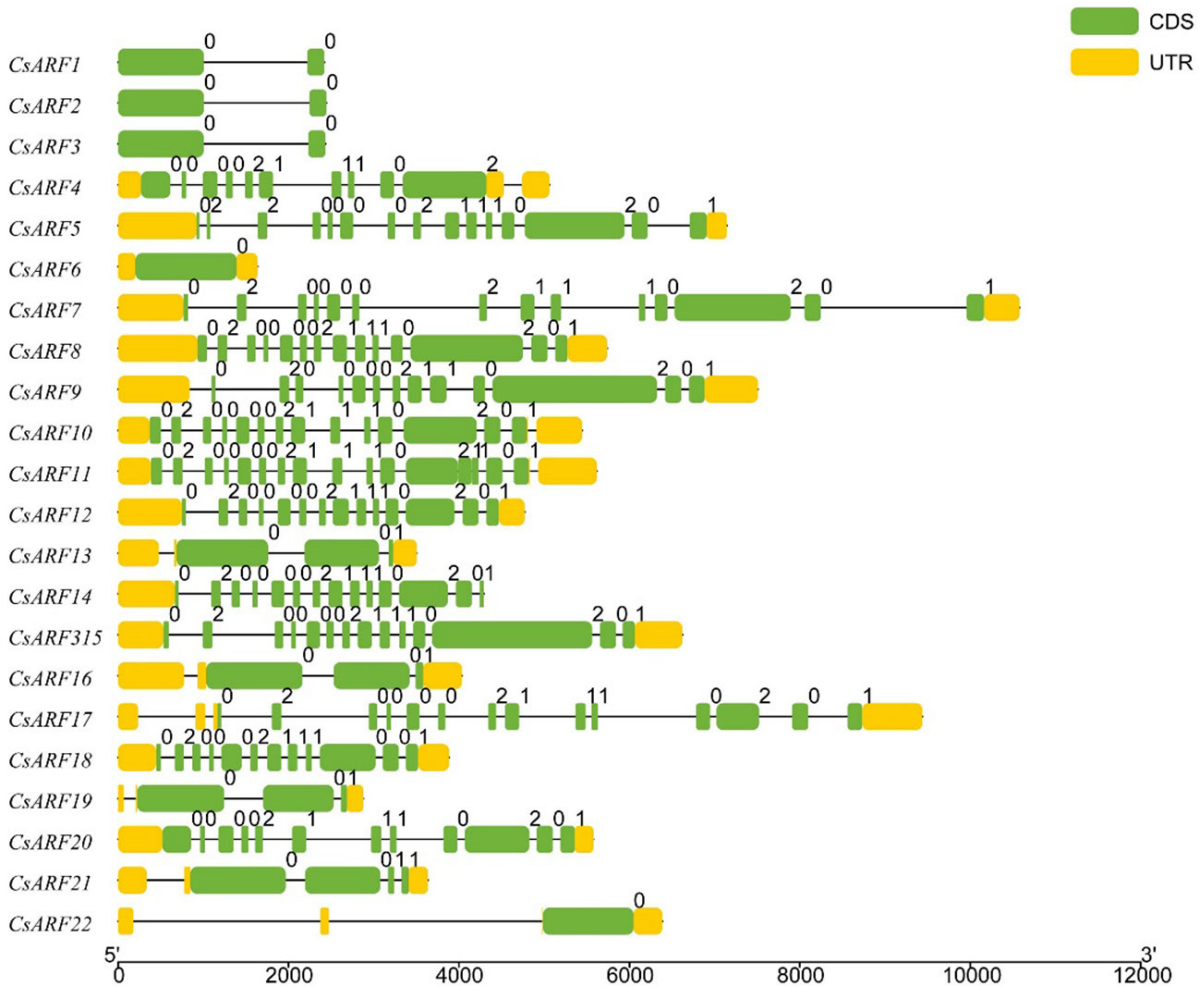
Gene structure analysis of cannabis *CsARF*. The exon, untranslated region (UTR), and intron are represented by yellow and green rectangles and a black line, respectively.

**Figure 2:**
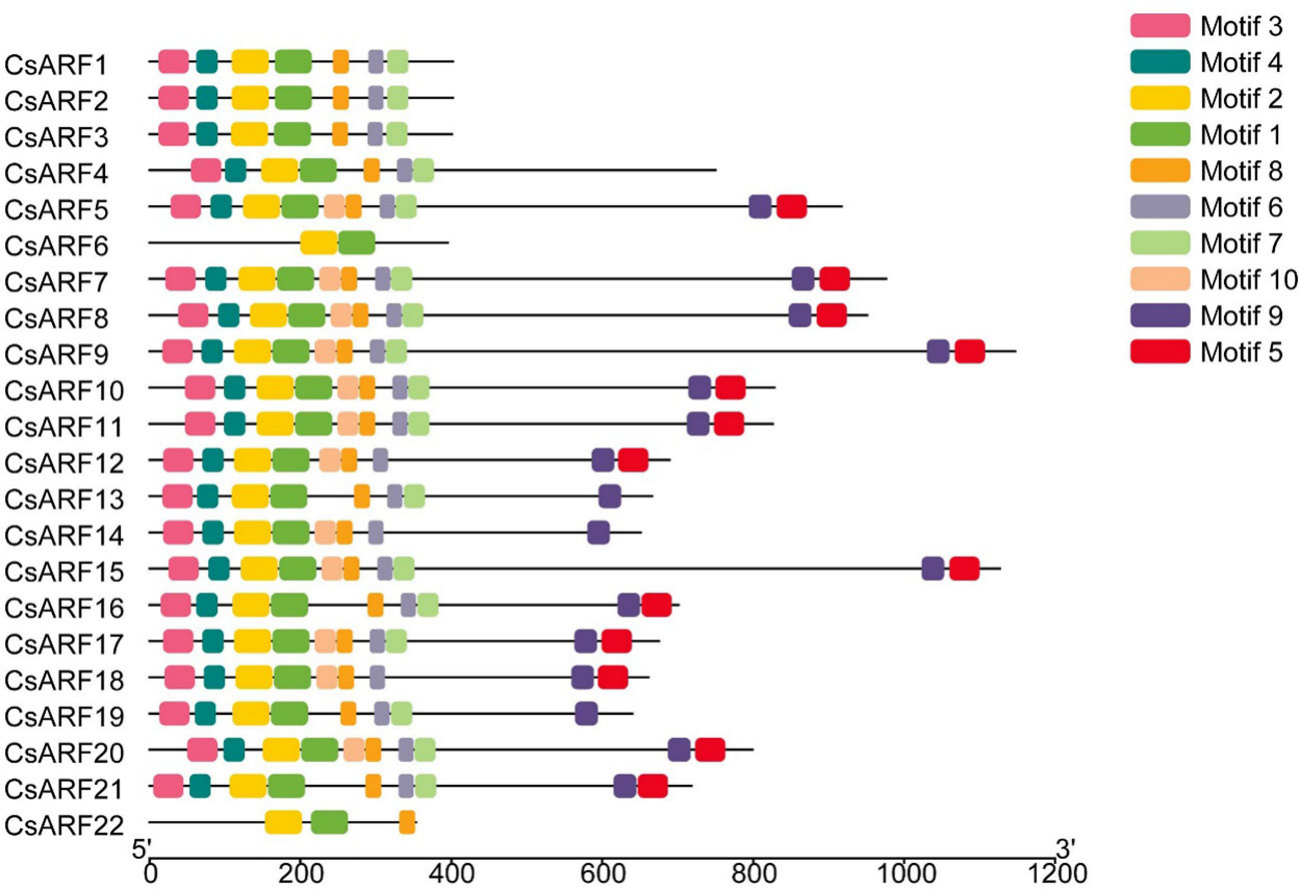
Motif distribution of cannabis CsARFs.

**Figure 3:**
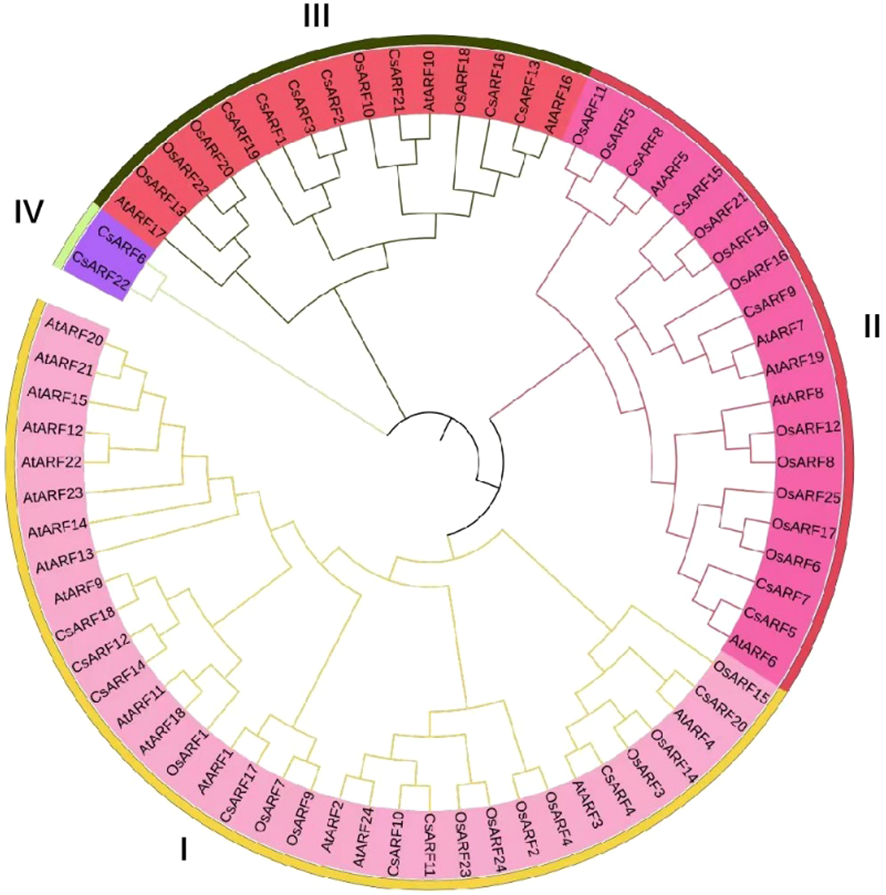
Phylogenetic tree of ARF proteins from three plant species. A phylogenetic tree was constructed based on the 90% shared amino acid sites using the neighbor-joining method. ARF, auxin response factor; At, *Arabidopsis thaliana*; Os, *Oryza sativa*; Cs, *Cannabis sativa.*

To investigate the structural diversity of *CsARF* genes, we analyzed the gene structure of *CsARF* using TBtool and MEME software. As shown in **Figure 1**, all *CsARF* genes except for *CsARF1*, *CsARF2* and *CsARF3* contained 3′ and 5′ UTR regions, and their proteins contained 1~15 introns and 2~16 exons. Motif analysis revealed that the number of motifs ranged from 2 to 10 (**Figure 2**). To analyze the evolutionary relationships between the *CsARF* genes of different species, a phylogenetic tree was constructed using ARFs from Arabidopsis and rice. A total of 69 ARF proteins were observed, including 44 genes from dicotyledonous plants (e.g., Arabidopsis and cannabis) and 25 from monocotyledonous plants (e.g., rice). As shown in **Figure 3**, the ARF proteins were grouped into four major subgroups: subgroups I, II, III, and IV. Subgroup I was the largest subfamily, containing 34 members, whereas subgroup IV contained only two members (**Figure 3**).

### 3.3 Analysis of Cis-regulatory elements in the promoter region of the *CsARF* gene family

**Figure 4:**
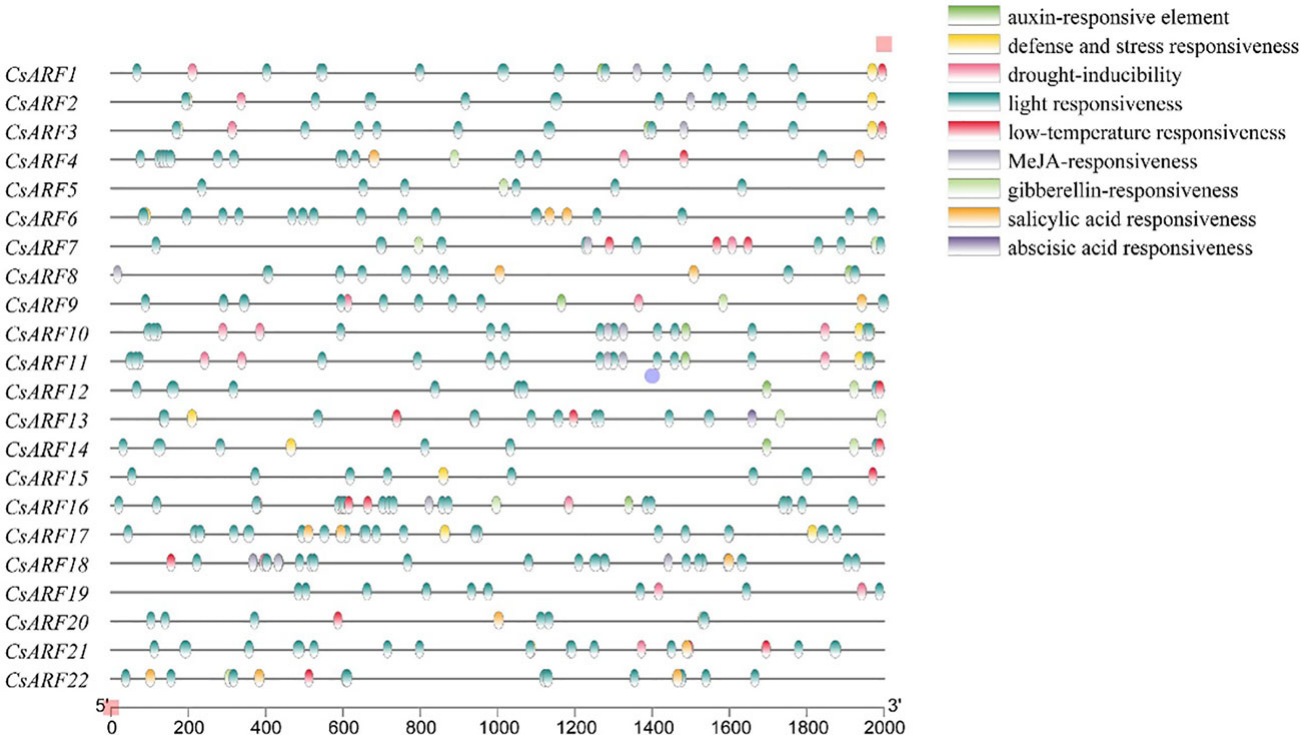
Analysis of *Cis*-regulatory elements in the upstream promoter of the *CsARF* genes.

*Cis*-regulatory elements located the promoter of a gene and acted as regulators of functional genes by binding to transcription factors. Therefore, the distribution of *Cis*-regulatory elements was further studied. As shown in **Figure 4**, nine different types of *Cis*-regulatory element were identified, among which 5 *Cis*-regulatory element related to hormones including auxin (IAA), Methyl Jasmonate (MeJA), gibberellin (GA), abscisic acid (ABA) and salicylic acid (SA), and light responsiveness was the main type of *Cis*-regulatory element in the promoter of *CsARF* gene. In addition, among the *CsARF* genes, *CsARF3* contains the most types of response elements (7), while *CsARF5* contains the least types (2) (**Figure 4**).

### 3.4 Chromosomal distribution and synteny analysis of cannabis *ARF* genes

**Figure 5:**
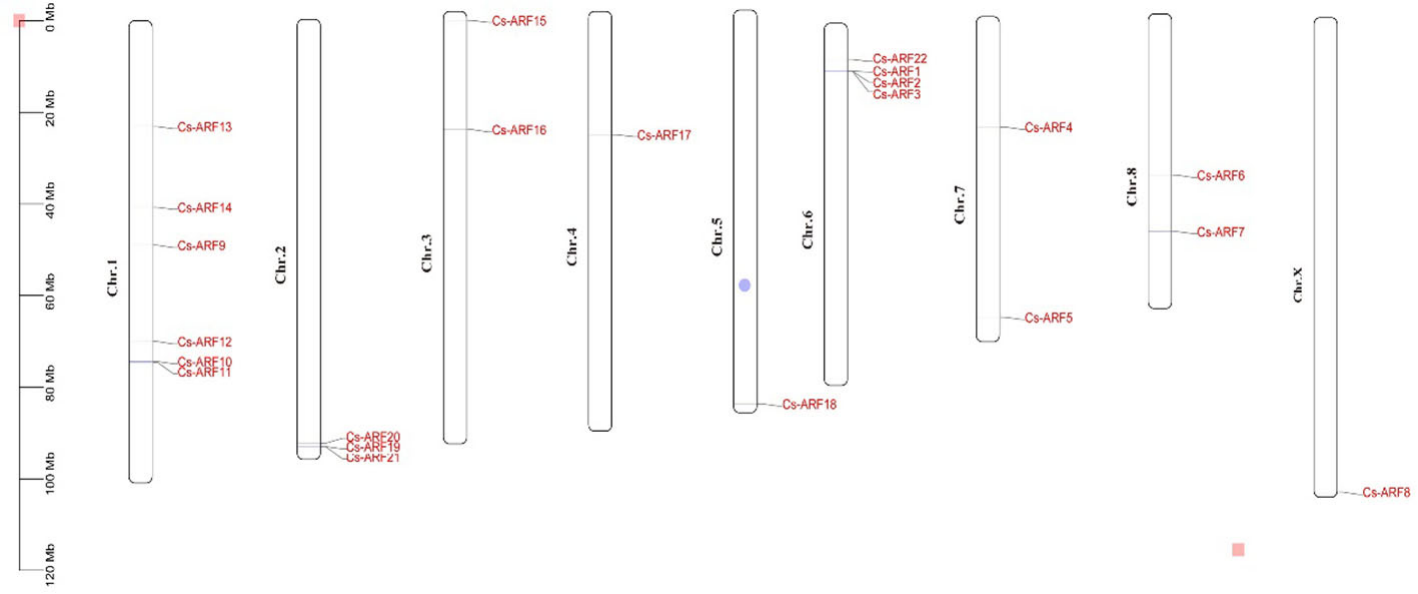
Physical locations of the 22 *CsARF* genes on cannabis chromosomes. The chromosome numbers are indicated on the left side of each scaffold. The chromosome size is shown on a vertical scale.

**Figure 6:**
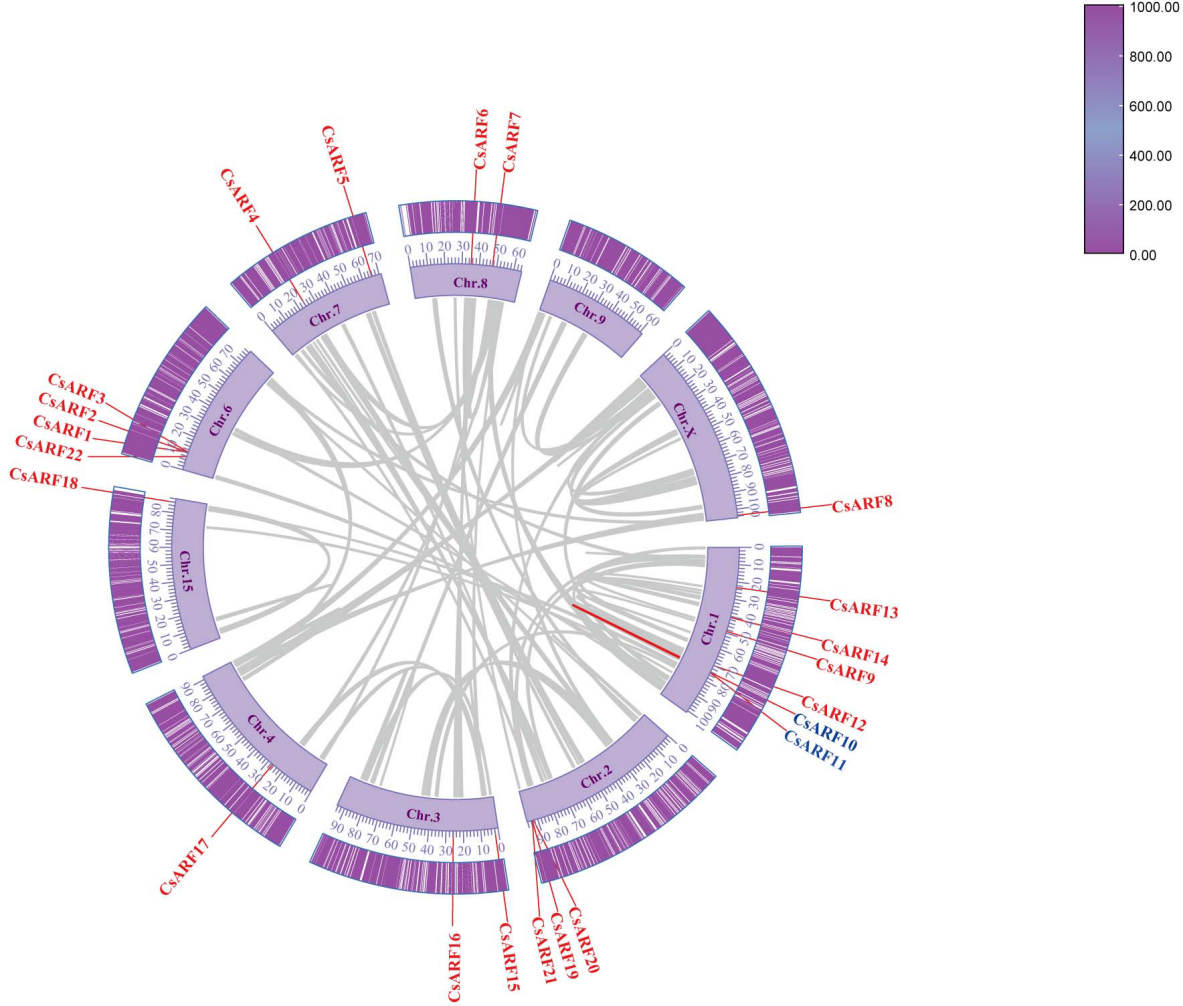
Schematic representation of the inter-chromosomal relationships between the *CsARF* genes in the cannabis genome. Colored lines indicates the colinear gene pair, red lines indicate the syntenic blocks in the cannabis genome.

**Figure 7:**
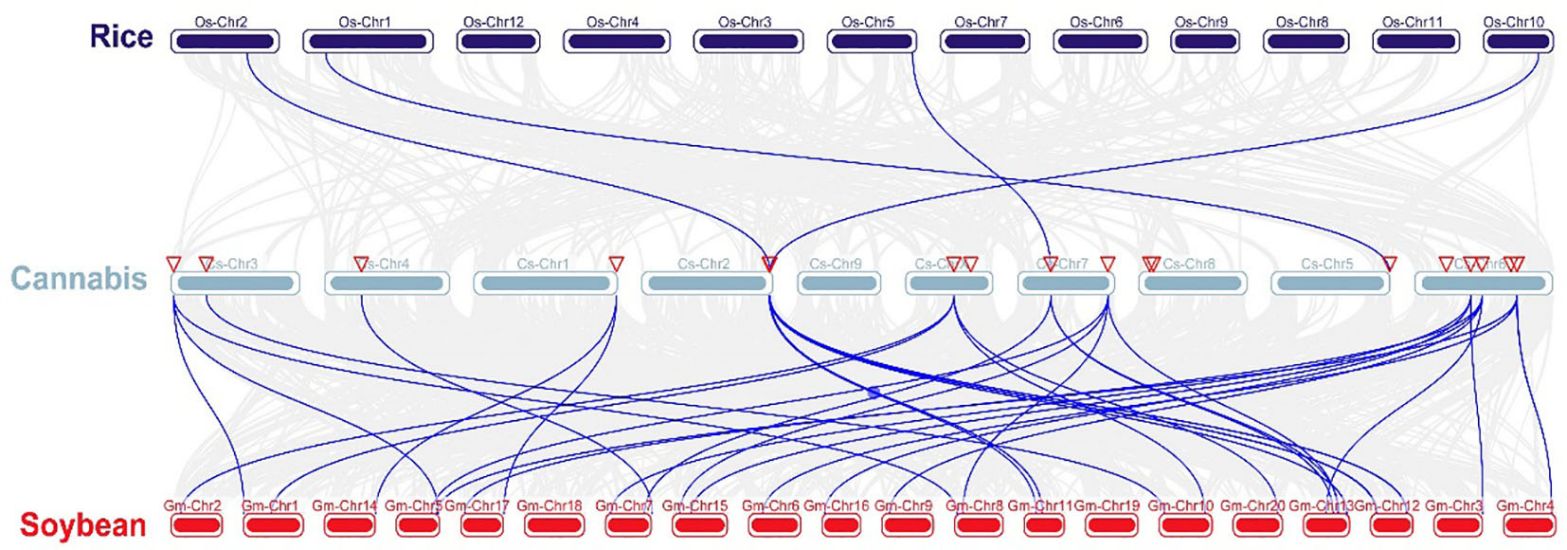
Synteny analysis of ARF genes between cannabis and two representative species. Gray lines indicate the syntenic blocks within the cannabis and other plant genomes. ARF is the auxin response factor.

Chromosomal distribution analysis revealed that 22 *CsARF* genes were unevenly distributed across the nine chromosomes of the cannabis genome, with the exception of chromosome 9 (**Figure 5**). Among these nine chromosomes, chromosome 1 contained the highest number of *CsARF* genes, while chromosomes 4, 5, and X contained only one gene (**Figure 5**). Three pairs of tandem duplication genes were identified on chromosomes 1 (*CsARF10/CsARF11*), 2 (*CsARF19/CsARF21*), and 6 (*CsARF1/CsARF2/CsARF3*), indicating that the expansion of the ARF family in cannabis was associated with tandem duplication events. Duplication events of *ARF* genes were also investigated in the cannabis genome. Only one pair of duplicated genes (*CsARF10*/*CsARF11*) was identified in the cannabis genome (**Figure 6**). To further understand the evolutionary mechanism of the ARF family in cannabis, collinearity diagrams of the ARF family from two dicotyledonous plants (soybeans and cannabis) and one monocotyledonous plant (rice) were constructed. As a result, 4 pairs of orthologous genes were identified between cannabis and rice (**Figure 7**), fewer than those identified in soybean and rice (29 pairs). Further, among these genes, the orthologs of two *CsARF* genes, *CsARF4* and *CsARF19*, were identified in both rice and soybean. *CsARF18* was only identified in rice, whereas 11 *CsARF* genes (*CsARF5*, *CsARF6*, *CsARF8*, *CsARF9*, *CsARF10*, *CsARF11*, *CsARF14*, *CsARF15*, *CsARF16*, *CsARF17* and *CsARF20*) were only present in soybean. The remaining genes were not identified in the duplicated blocks (**Figure 7**).

### 3.5 Subcellular localization analysis of CsARF2

**Figure 8:**
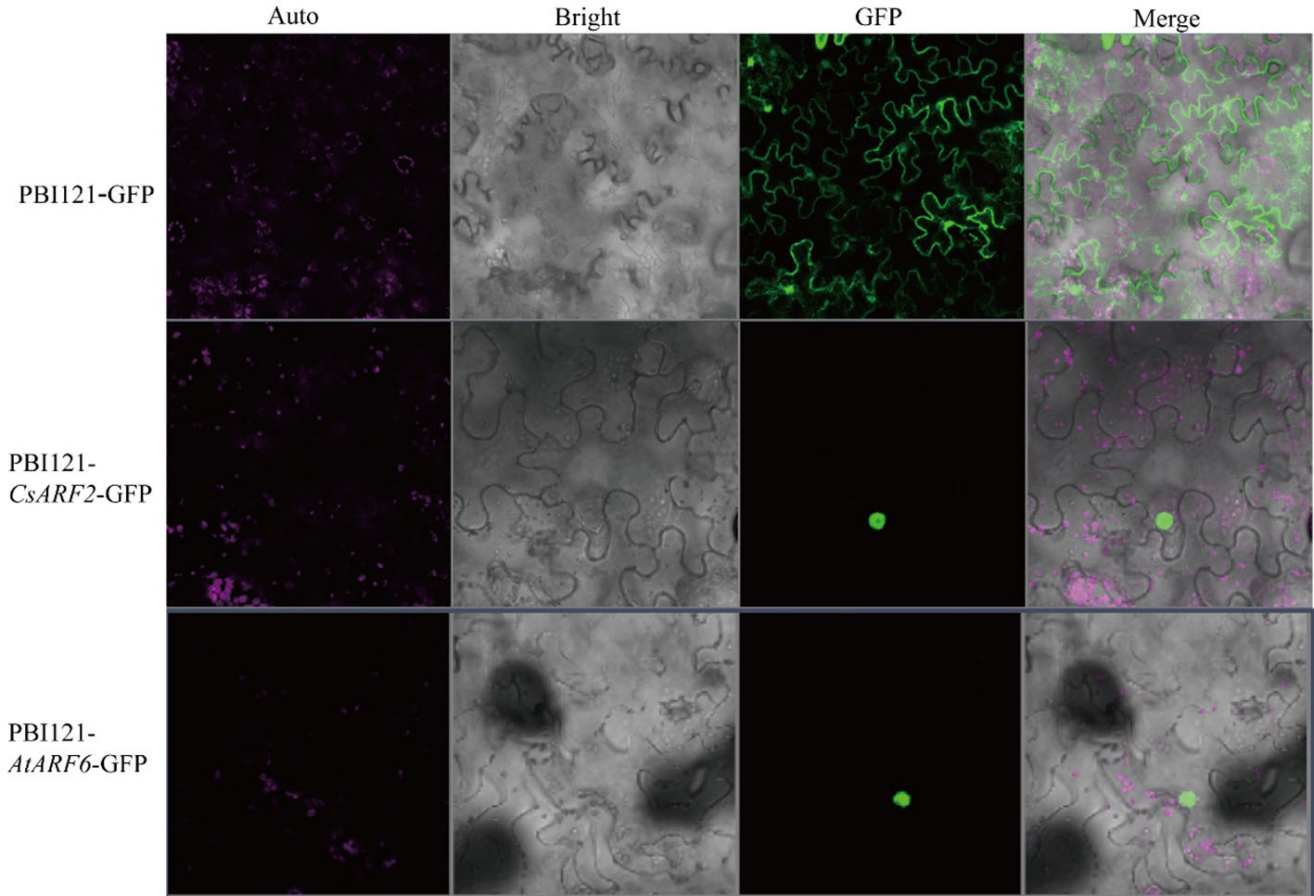
Subcellular localization analysis of CsARF2 in tobacco leaves. A AtARF6 was used as the positive control.

Subcellular localization predication of the CsARFs was performed, and the results showed that most of the CsARF proteins were localized in the nucleus, except for the single protein CsARF22, which localized in the cytoplasm (**Table 1**). To verify the results of subcellular localization predication, CsARF2 was then randomly selected for subcellular localization assay in tobacco leaves. As shown in **Figure 8**, CsARF2 was localized in the nucleus of tobacco leaf cells compared with the control (the empty vector pBI121-GFP) and the positive control (AtARF6 in Arabidopsis). These results indicate that *CsARF2* functions as a transcriptional regulator in the nucleus, and may involve in the regulation of growth and development in cannabis.

### 3.6 Expression profiling of *CsARF* genes in different tissues and at different developmental stages of female flower

**Figure 9:**
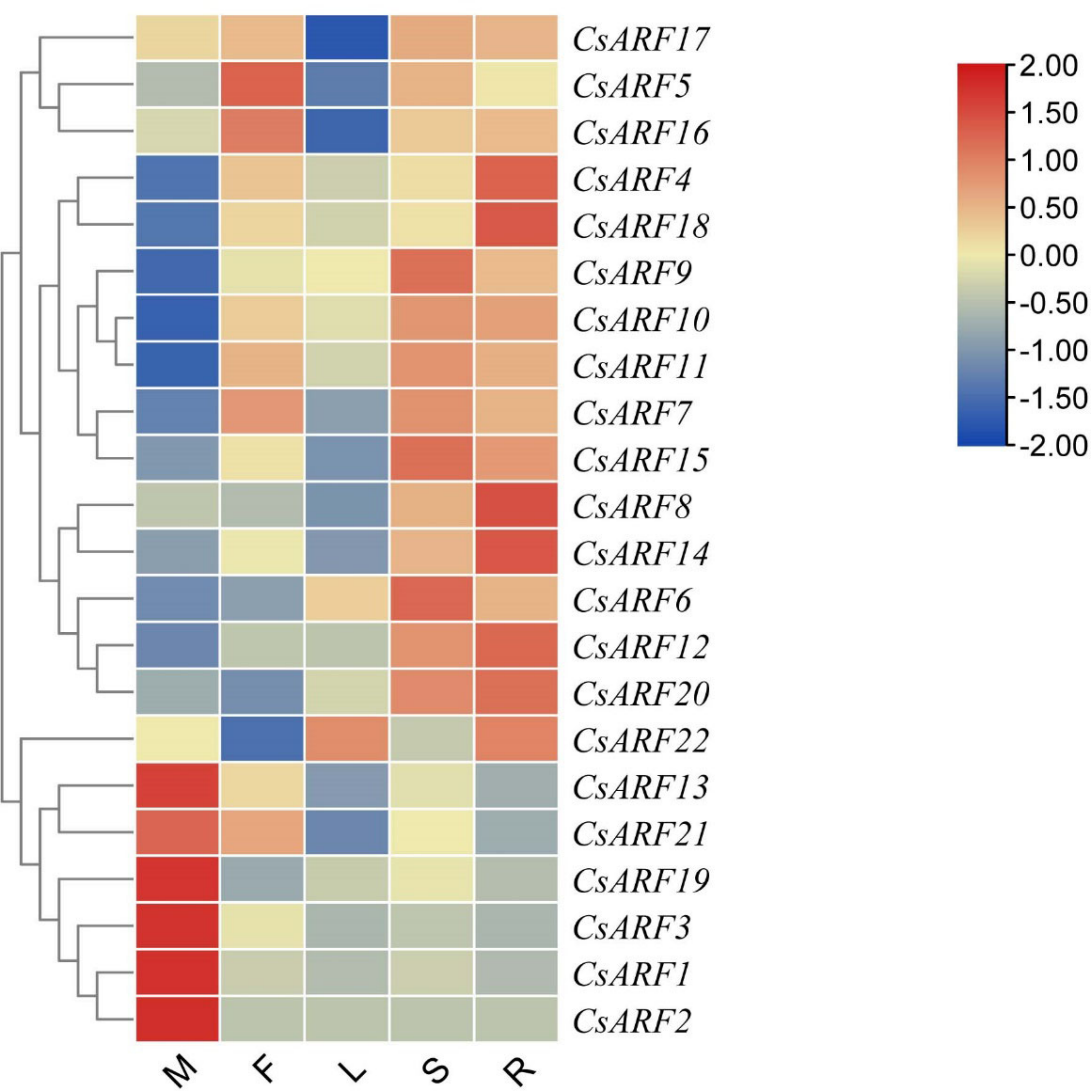
Tissue-special expression analysis of *CsARF* genes in cannabis ‘USO14’ monoecious cultivars. M: male flower; F: female flower; L:leaf; S:stem; R:root.

To explore the potential role of *CsARF* genes in cannabis development, the expression profile of *CsARF* genes was analyzed in five tissues obtained from the transcriptome data of ‘USO14’ monoecious cultivars in our laboratory (unpublished). As a result, 22 *CsARF* genes exhibited differential expression patterns in different tissues. Six *CsARF* genes, including *CsARF1*, *CsARF2*, *CsARF3*, *CsARF13*, *CsARF19* and *CsARF21*, were preferentially expressed in male flowers; *CsARF5* and *CsARF16* were highly expressed in female flowers; five *CsARF* genes (*CsARF6*, *CsARF7*, *CsARF9*, *CsARF11* and *CsARF15*) showed high expression levels in the stem; and the transcript levels of *CsARF4* and *CsARF8* were higher in the roots than those in the other four tissues (**Figure 9**).

**Figure 10:**
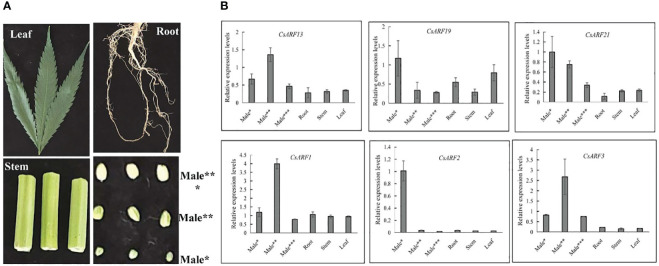
Expression profiling of six *CsARF* genes in different tissues and male flower development stages. **(A)**: Representative images of different tissues and male flower development stages of cannabis plant. Male*: The length was about 1 mm, the anthers and pollen grains were green; Male**: The length was about 2 mm, the anthers was green, and pollen grains were yellow; Male***: The length was about 3–4 mm, the anthers and pollen grains were yellow; **(B)**: transcript levels of six *CsARF* genes. ARF is the auxin response factor.

To gain insight into the role of *CsARF* genes in male development, we investigated the expression patterns of the six genes with the highest transcript levels at different stages of male flower development (**Figure 10A**). As shown in **Figure 10B**, three *CsARF* genes including *CsARF3*, *CsARF13* and *CsARF21*, were found to be highly expressed at all female flower stages compared to the other four tissues, suggesting that these genes may be involved in the female flower development.

### 3.7 Expression patterns of *CsARF* genes in the male and female tissue of two varieties with distinct CBD content

**Figure 11:**
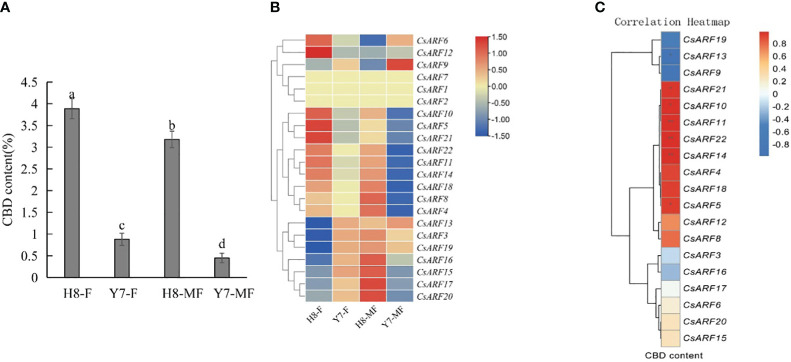
Correlation analysis between the expression level of *CsARF* genes and CBD content in male and female flower tissues of two cultivars ‘H8’ and ‘Y7’. **(A)**: Determination of CBD content in female and male flowers in two cannabis cultivars; **(B)**: Heat map of the expression levels of 22 *CsARFs* in female and male flowers in two cannabis cultivars, ‘H8’ and ‘Y7’. Red and blue indicate high and low transcript abundances, respectively. **(C)**. Correlation heatmap between gene expression of *CsARF* gene and CBD content in female flower and male flower of ‘H8’ and ‘Y7’ based on the Pearson’s correlation coefficient. Three independent samples were used for expression analysis. H8-F, female flower of ‘H8’; H8-MF, male flower of ‘H8’; Y7-F, female flower of ‘Y7’; Y7-MF, male flower of ‘Y7’. CBD is cannabidiol. Significant differences were determined using one-way ANOVA, Different letters indicate significant difference at *P* < 0.05. *P<0.05; **P<0.05.

To investigate the possible role of *CsARF* genes in the regulation of CBD biosynthesis, the expression levels of all *CsARF* genes in female and male cannabis varieties with high CBD content ‘H8’ and low CBD content ‘Y7’ were detected by qRT-PCR (**Figure 11A**). The CBD content of female and male tissues of ‘H8’ was higher than that of ‘Y7’ (**Figure 11A**). Eleven genes were more highly expressed in ‘H8’ female flowers (H8-F) than in Y7 female flowers (Y7-F), and 15 genes showed higher transcript levels in ‘H8’ male flowers (H8-MF) than in Y7 male flowers (Y7-MF) (**Figure 11B**). Furthermore, correlation analysis between the expression level of *CsARF* genes and CBD content in male and female flower tissues of two cultivars ‘H8’ and ‘Y7’ showed that the expression level of *CsARF13* was negatively correlated with CBD content, while the expression levels of six genes (*CsARF5*, *CsARF10*, *CsARF11*, *CsARF14*, *CsARF21* and *CsARF22*) were positively correlated with CBD content (**Figure 11C**).

### 3.8 Expression analysis of *CsARF* genes in response to IAA

**Figure 12:**
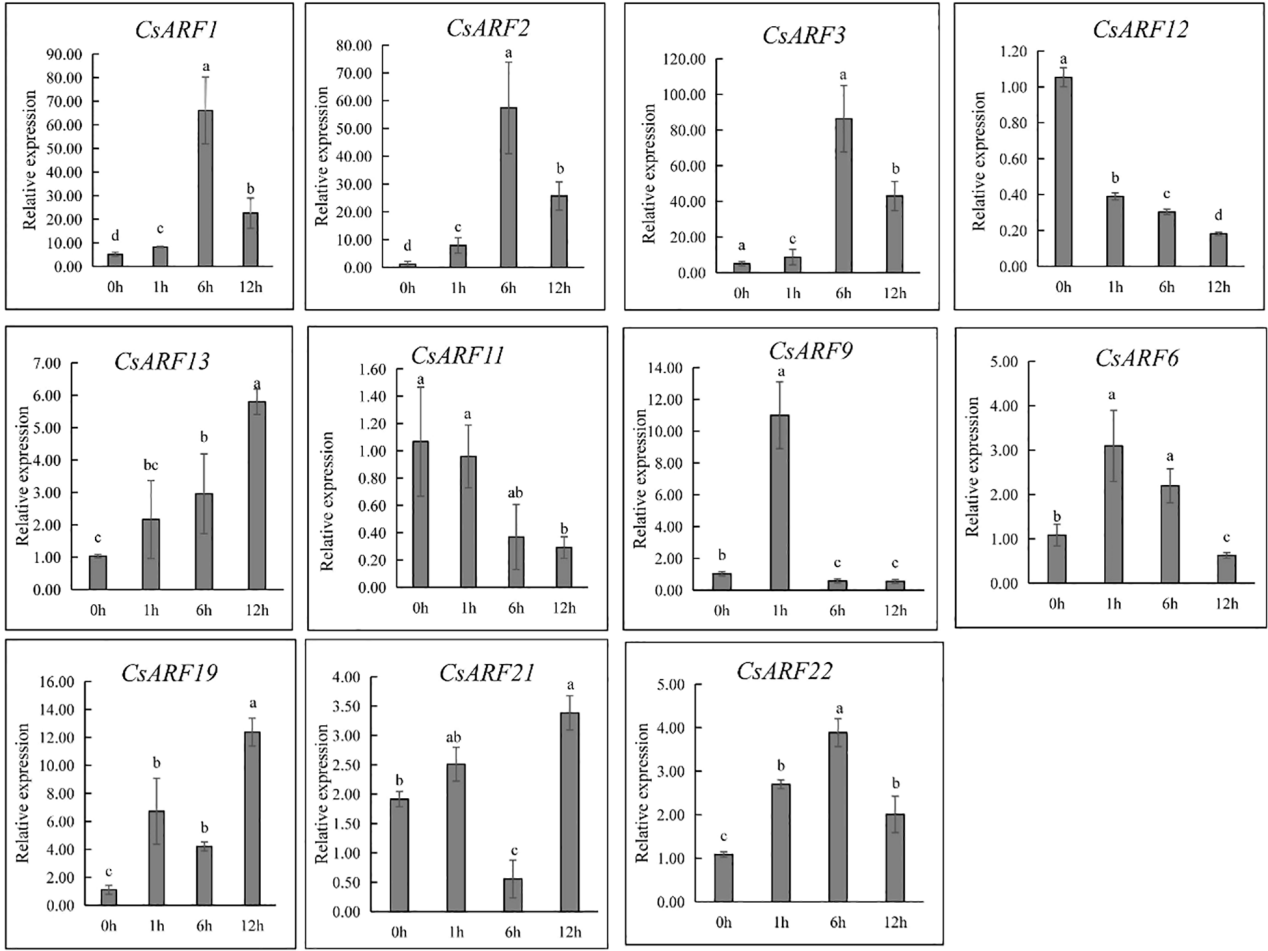
Expression level analysis of 11 genes response to 20 µM of IAA treatment at different points post IAA treatment. Different letters indicate significant difference at *P <* 0.05. IAA is indole-3-acetic acid.

ARFs are important regulators of auxin signaling as they are sensitive to auxin. To investigate whether ARFs respond to auxin, cannabis ‘H8’ leaves were treated with IAA and used for gene expression analysis at 0, 1, 6 and 12 h. Half of all *CsARF* genes were randomly selected for qRT-PCR analysis. As a result, nine genes were induced by IAA treatment except for *CsARF11* and *CsARF12* (**Figure 12**). However, their expression patterns were significantly different. *CsARF6* and *CsARF9* were strongly induced at 1 h after IAA treatment, and the expression of *CsARF1*, *CsARF2, CsARF3* and *CsARF22* increased at 1 h and peaked at 6 h after IAA treatment, whereas the transcript levels of the remaining IAA-induced genes peaked at 12 h after IAA treatment (**Figure 12**).

## 4 Discussion

The *ARF* gene family plays an important role in regulating flower development and secondary metabolite production and has been studied in many plant species, including Arabidopsis (Okushima et al., 2005), rice (Wang et al., 2007), *Euscaphis konishii* (Liu et al., 2022), and maize (Xing et al., 2011). However, a comprehensive investigation of the *ARF* gene in cannabis has not yet been conducted. In this study, 22 ARF members were identified in the cannabis genome (**Table 1**), and the number is lower than that of rice (25) (Wang et al., 2007) and maize (36) (Xing et al., 2011), similar to that of tomato (22) (Kumar et al., 2011) and Arabidopsis (23) (Okushima et al., 2005). Considering that these plants have different genome sizes and *ARF* numbers, the number of *ARF* members was not stable, probably due to gene deletion and expansion in different species.

Duplication events, including tandem duplication and segmental/whole duplication, are the major forces for gene family expansion and functional differentiation during evolution. Previous studies have reported that plant *ARF* genes expand by undergoing tandem duplication and segmental/whole duplication (Chen et al., 2023; Qi et al., 2023). For example, four *LuARF* genes (two gene pairs) underwent tandem duplication events, whereas 27 (81.8%, 18 gene pairs) underwent segmental/whole duplication events in flax (Qi et al., 2023). In alfalfa, 14 segmental duplications and two sets of tandem repeats were identified (Chen et al., 2023). Similarly, seven *CsARF* genes (three sets of gene pairs) underwent tandem duplication, and one segmental duplication was identified (**Figures 5**, **6**, 7). These findings indicate that the evolution of *ARF* gene is conserved, mainly due to tandem and segmental/whole duplication. Consistent with the findings of this study, duplications were also found to occur in the *CBCAS*, *THCAS*, *CBDAS*, *COL*, and *MYB* genes in cannabis (Weiblen et al., 2015; Laverty et al., 2019; Pan et al., 2021; Yin et al., 2022). Further, two pairs of duplicated genes (*CsARF19/CsARF21* and *CsARF1/CsARF2/CsARF3*) displayed similar expression patterns in five cannabis tissues (**Figure 9**), but showed distinct expression profiles in female and male flowers of two varieties with different CBD content (**Figure 11**), suggesting that these genes may undergo functional divergence in the regulation of CBD production during gene duplication. In addition, four pairs of orthologous genes were identified between cannabis and rice, whereas 29 pairs were found between soybean and rice (**Figure 7**). These findings imply that cannabis *ARF* genes have a closer relationship with soybean than rice, which may be consistent with the evolutionary relationship between monocotyledons and dicotyledons. *CsARF4* and *CsARF19* were identified in both rice and soybean, indicating that the expansion of these *ARF* genes occurred in a species-specific manner from common ancestral genes prior to the dicot-monocot divergence.

In cannabis CBD production, increasing the CBD content of cannabis plants and reducing the negative effect of male plant pollination on CBD effectively increased the CBD yield. Thus, the identification of candidate genes involved in the regulation of male development and CBD biosynthesis could provide genetic resources for increasing CBD yield through genetic modification. In this study, we identified two *CsARF* candidate genes, *CsARF10* and *CsARF11*, whose expression levels showed a positive correlation with CBD content and were lowest in male flowers compared to four other tissues (**Supplementary Figure S1**, **Figure 11**), suggesting that these genes positively regulate CBD biosynthesis and negatively regulate male flower development (**Figures 9**, **10**, **11**). In contrast, only *CsARF13* showed low expression levels in varieties with high CBD content and high expression levels in male flowers, suggesting that it may act as a positive regulator of male flower development and a negative regulator of CBD biosynthesis. There, to increase CBD biosynthesis and enhance the CBD yield, we can achieve this goal by overexpressing four *CsARFs* (*CsARF4*, *CsARF11* and *CsARF18*) and by gene editing of *CsARF13* in the future.

Auxins play an important role in plant development and secondary metabolite biosynthesis, and their signaling pathway is mediated by ARF proteins. In this study, 9 of the 11 *CsARF* genes were induced by IAA treatment. Among the nine genes, the transcript levels of *CsARF3*, *CsARF13* and *CsARF21* were highly expressed in male flowers at different stages (**Figures 9**, **10**). However, previous studies have shown that IAA inhibits the formation of male flower (Galoch, 1978). Thus, we hypothesized that *CsARF3*, *CsARF13* and *CsARF21* regulate male flower development in an IAA-independent manner, which requires further investigation. In addition, IAA is involved in the cannabinoid biosynthetic pathway by activating the expression of *CsPT1*, a key enzyme that catalyzes the alkylation of OA and GPP to form CBGA, the precursor of CBD (Sands et al., 2023). Meanwhile, the promoter of *CsPT1* was found to contain an AuxRE and an ARF binding site (Sands et al., 2023), and our findings revealed that candidate regulators of CBD biosynthesis, *CsARF21* and *CsARF22*, are induced by IAA treatment. Taken together, these results suggest that IAA affects cannabinoid biosynthesis via the induction of *CsARF21* and *CsARF22*, which may bind to the AuxRE of *CsPT1* to regulate its expression.

## 5 Conclusions

In this study, a systematic analysis of the ARF family was performed in cannabis. A total of 22 *CsARF* were identified and divided into four subgroups. The gene location, gene structure, conserved motifs, gene duplication, subcellular localization and *cis*-acting element characteristics of CsARFs were then investigated. Moreover, the expression patterns of *CsARFs* in different tissues, different varieties and the responses to IAA treatment were investigated. These genes were unevenly distributed on nine chromosomes and duplication events occurred in cannabis during evolution. Subcellular localization assay showed that CsARF2 were localized in nucleus. *CsARF3*, *CsARF13* and *CsARF21* were highly expressed in male flowers at different developmental stages and were induced by IAA treatment. In addition, the transcript levels of nine *CsARFs* showed similar trends in CBD variation in the male and female flowers of the two cannabis varieties. These findings reveal the potential roles of *CsARFs* in male flower development and CBD biosynthesis, and provide gene resources for molecular breeding for high CBD yield.

## Data availability statement

The accession numbers for the cannabis genome, protein, and genome annotation files used in this study was SAMEA5040675 in NCBI database. The primer sequence used in this study can be found in the article/**Supplementary Material**.

## Author contributions

GP: Funding acquisition, Methodology, Writing – original draft. XY: Project administration, Writing – original draft. JH: Formal analysis, Writing – original draft. ZL: Data curation, Writing – original draft. FC: Funding acquisition, Methodology, Writing – review & editing. JC: Supervision, Writing – review & editing.
