# Supplementary material for: Comprehensive analyses of the ARF gene family in cannabis reveals their potential roles in regulating cannabidiol biosynthesis and male flower development
Source: Front Plant Sci. 2024 Jun 5;15:1394337. doi: 10.3389/fpls.2024.1394337 (PMC11188406; doi:10.3389/fpls.2024.1394337)
Supplement: Supplementary file 1 [file DataSheet_1.doc]

Table S1 Primers used for in this study

| Usage | Gene | Forward primer | Reverse primer |
| --- | --- | --- | --- |
| qRT-PCR | CsARF1 | TGAGGTGGGATGTTGATGCT | CACGTTTGCGGTCATAGGTT |
|  | CsARF2 | AGCCAAGCAAGTGGTTTCTG | ATTCTTCCTTGCCACCTTGC |
|  | CsARF3 | TTCCTCCGGTCAACTCTACG | ACTCGGCATTGAATCCTCCA |
|  | CsARF4 | GTGCCAAGCTCTAGAAGTGC | GTAGCTCTCTGCTCCTCCTG |
|  | CsARF5 | CCTTGGCATGAACTCTCAGC | GTGAGATCCCAACACCCTGA |
|  | CsARF6 | CCACAACAACCGCATGATGA | CGCGTGCTGTTTGGGTATAA |
|  | CsARF7 | AGCATCAGCAGCAACATCAG | AGCGATGGAGACTGAGACTG |
|  | CsARF8 | GCAAGCCTAGGTGACTCTCA | TTCGCACTGGTGGAACTACT |
|  | CsARF9 | AGGTCCCAGTTTCCAACCAA | TCCTCGTACTGTCCTCTCCA |
|  | CsARF10 | CCTCCAACTCAGGAACTGGT | CCAGCCACTTTGAAGCAAGT |
|  | CsARF11 | CCTCCAACTCAGGAACTGGT | CCAGCCACTTTGAAGCAAGT |
|  | CsARF12 | CACTGAATGCTTGCCTCCTC | CCTTCTCGGTTGGCCTCTAA |
|  | CsARF13 | CGTTGGCCTGATTCTCCTTG | GTGGAAGCCGCATCTTCTTT |
|  | CsARF14 | CACTGAATGCTTGCCTCCTC | CCTTCTCGGTTGGCCTCTAA |
|  | CsARF15 | GCTCAGCAGGAGCTTTCATC | AGCTGCCATCGTTGATTGTC |
|  | CsARF16 | TTGGAGGAGGGCTTAATGGG | GGAAAGATGGTTTCGGCACA |
|  | CsARF17 | TTGCTCACTACTGGGTGGAG | CTAACTCCAACACGCAGCTC |
|  | CsARF18 | GATAGAGTGTCGCCTTGGGA | TTCCAGACTGCTGATGTGGT |
|  | CsARF19 | CCTCGTTCTCTATGGCGAGT | GAGCAAGAGTTCATGCGTGT |
|  | CsARF20 | GGGATGCAGTGCTCTTTCTG | CATGAGTTGCCCTTGGACTG |
|  | CsARF21 | GTTGTGGTGGCAATTCTGGT | ACCGAAGAGGCCTTAACACA |
|  | CsARF22 | ACGGTTGATGACATGGAGGT | TGGTGTCATTTGCCTTGAGC |
| Subcellular localization | CsARF2 | acgggggactctagaggatccATGAACGAAGAAAGAGTTCAGATAATCG | gcccttgctcaccatggatccTCCTGATCTCCTCTTGGATTCTCC |
|  | AtARF6 | acgggggactctagaggatccATGAGATTATCTTCAGCTGGGTT | gcccttgctcaccatggatccAGTTGGGGGTTCATTCAAC |
|  |  |  |  |


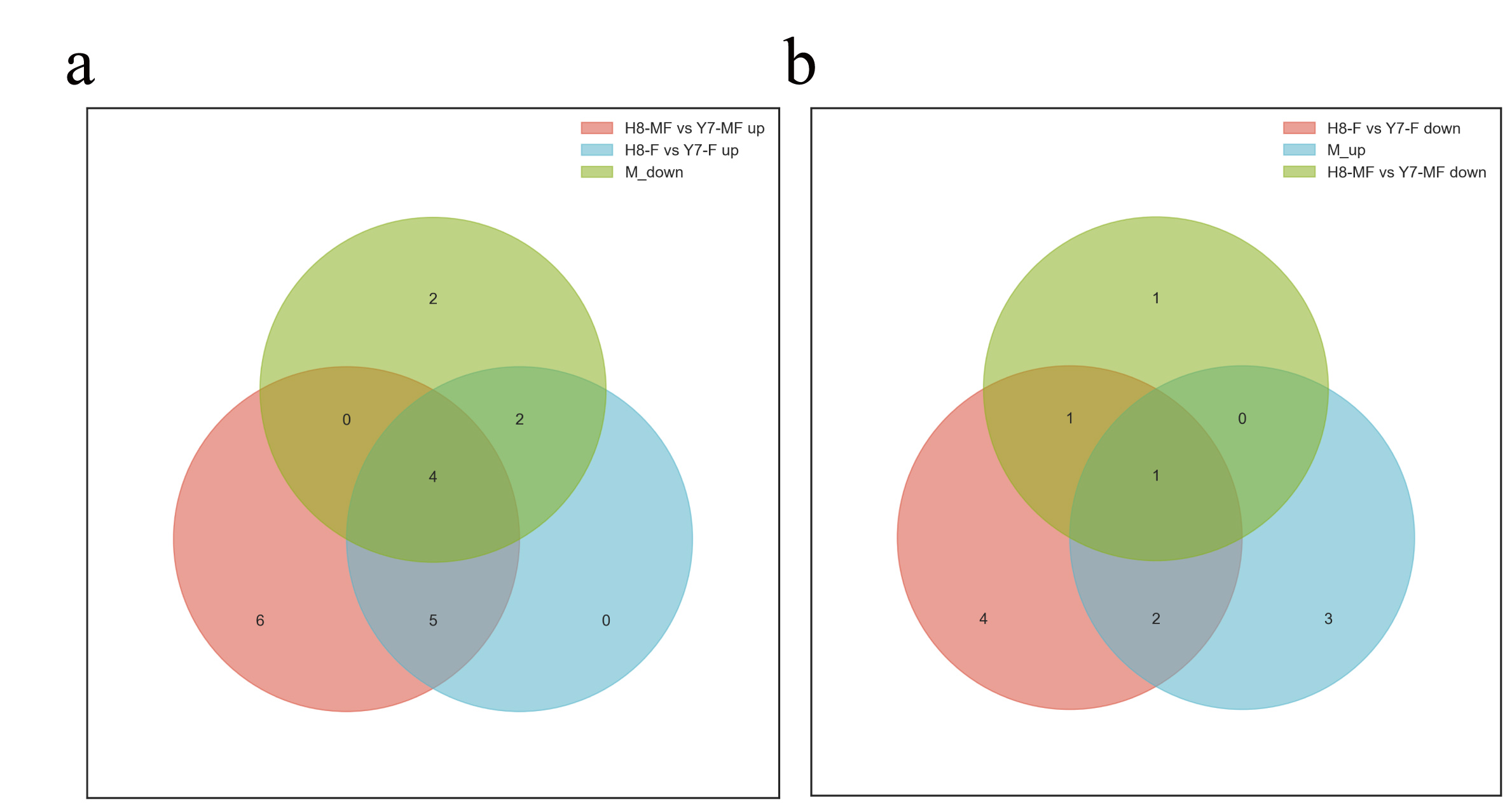


**Figure S1** Venn diagram showing the distribution of DEGs in the different groups “H8-MF vs Y7-MF”, “H8-F vs Y7-F” and “M vs four other tissues” (root, female flower, stem, leaf).

H8-F, female flower of H8; H8-MF, male flower of H8; Y7-F, female flower of Y7; Y7-MF, male flower of Y7. M_up: The highest expression levels of genes in the male flower compared to four other tissues including root, female flower, stem and leaf. M_down: The lowest expression levels of genes in male flower compared to four other tissues including root, female flower, stem and leaf.
